# Biocapture of CO_2_ by Different Microalgal-Based Technologies for Biogas Upgrading and Simultaneous Biogas Slurry Purification under Various Light Intensities and Photoperiods

**DOI:** 10.3390/ijerph15030528

**Published:** 2018-03-15

**Authors:** Pengfei Guo, Yuejin Zhang, Yongjun Zhao

**Affiliations:** 1 Qin Tan (Shanghai) Environmental Engineering Co. Ltd., Shanghai 200232, China; xujie@mail.zjxu.edu.cn; 2 College of Biological Chemical Science and Engineering, Jiaxing University, Jiaxing 314001, China

**Keywords:** biogas upgrading, co-cultivation, CO_2_ removal, light intensities, photoperiods

## Abstract

Co-cultivation of microalgae and microbes for pollutant removal from sewage is considered as an effective wastewater treatment method. The aim of this study is to screen the optimal photoperiod, light intensity and microalgae co-cultivation method for simultaneously removing nutrients in biogas slurry and capturing CO_2_ in biogas. The microalgae–fungi pellets are deemed to be a viable option because of their high specific growth rate and nutrient and CO_2_ removal efficiency under the photoperiod of 14 h light:10 h dark. The order of both the biogas slurry purification and biogas upgrading is ranked the same, that is *Chlorella vulgaris*–*Ganoderma lucidum* > *Chlorella vulgaris*–activated sludge > *Chlorella vulgaris* under different light intensities. For all cultivation methods, the moderate light intensity of 450 μmol m^−2^ s^−1^ is regarded as the best choice. This research revealed that the control of photoperiod and light intensity can promote the biological treatment process of biogas slurry purification and biogas upgrading using microalgal-based technology.

## 1. Introduction

Owing to fossil fuel combustion during anthropogenic activities, greenhouse gas (GHG) emissions are ever increasing at present, which induces an increased interest in searching for renewable, sustainable and environment-friendly energies as an alternative to fossil fuel due to increased concern over the energy crisis, global warming and climate changes [1]. Biogas is one of the most important renewable energy resources and has attracted the most attention in both developed and developing countries in recent years [2]. Raw biogas mainly consists of methane (CH_4_, 40–75%, *v*/*v*), carbon dioxide (CO_2_, 15–60%, *v*/*v*), and trace amounts of vapor (H_2_O, 5–10%, *v*/*v*), oxygen (O_2_, 0–1%, *v*/*v*), and hydrogen sulfide (H_2_S, 0.005–2%, *v*/*v*) [3]. Obviously, the presence of relatively high concentration of CO_2_ greatly reduces the enthalpy and calorific value of crude biogas. Therefore, CO_2_ should be removed to enhance CH_4_ concentration to meet the efficient combustion standard (CH_4_ > 90%, *v*/*v*) [4]. Chemical absorption, pressure swing adsorption, membrane separation, cryogenic separation, and water scrubbing are the most frequently-used biogas upgrading technologies [5,6]. However, they usually face some severe challenges and obstacles, such as high capital cost for construction, large amount of energy during treating process, complicated operating systems, and/or unwanted end products that need further treatment or result in secondary pollution. Furthermore, during the process of biogas upgrading, CO_2_ is always discharged into the atmosphere as greenhouse gas in these conventional techniques [7,8]. Accordingly, photosynthetic CO_2_ uptake by microalgae is an alternative technique to upgrade biogas, as they have high carbon fixation capacity, rapid specific growth rate, and strong environmental adaptability. In addition, biogas slurry could provide most of the required nutrients for microalgae growth as a readily available nutrient medium [9]. Therefore, removing CO_2_ from raw biogas by culturing microalgae in biogas slurry is a highly prospective technique for simultaneous biogas upgrading and biogas slurry decontamination, characterized as renewable, sustainable and environment-friendly energy.

Recently, mono-cultivation of microalgae has already been successfully utilized for simultaneous biogas slurry treatment and CH_4_ enrichment based on several crucial factors, such as microalgae species, light wavelength, light intensity, initial influent CO_2_ concentration, light photoperiod, etc. [1,2,10,11,12]. For instance, Yan and Zheng [13] reported that components of upgraded biogas content (*v*/*v*) were 93.68 ± 3.25% CH_4_, 1.57 ± 0.42% CO_2_, and the highest removal efficiencies of chemical oxygen demand (COD), total nitrogen (TN) and total phosphorus (TP) of biogas slurry were 78.91 ± 2.75%, 73.05 ± 2.04%, and 67.54 ± 1.46%, respectively. However, harvesting of the microalgae cell from industrial cultivation for biofuel production, wastewater treatment or value-added chemicals cultivation have always been the major obstacles for the algae-to-fuel approach, as the addition of chemicals or excessive energy demand were required [14,15]. To resolve these mentioned challenges, bio-flocculation has been widely applied by employing suitable microbial partner, such as algal–algal, algal–fungal and algal–bacterial interactions [15,16]. It has been shown that bacteria and fungi could bio-flocculate with microalgae. In submerged cultures, the bio-flocculation could aggregate and grow into granules or pellets [14]. For co-cultivation of microalgae and fungi, pelletization of filamentous fungi with microalgal biomass has been recently reported as an efficient algal harvesting technique, which is commonly observed in the fungal fermentation process [17,18,19]. As far as co-cultivation with microalgae and bacteria was concerned, Sun et al. (2016) reported that significant effect on biogas upgrading and simultaneous biogas slurry nutrients reduction has been found using typical cooperative algal–bacterial systems (microalgae *Chlorella vulgaris*, *Scenedesmus obliquus* and *Neochloris oleoabundans* mixed with activated sludge) and high levels of biomass productivity were also observed in the experiment [20]. Similarly, our previous research already focused on three typical treatment technologies (i.e., mono-cultivation of microalgae, co-cultivation of microalgae and fungi, and co-cultivation of microalgae and activated sludge), and demonstrated that both algal–fungal and algal–bacterial symbiosis could effectively perform the simultaneous biogas upgrading and biogas slurry purification based on the constant light intensity and stationary photoperiod in photobioreactor [3]. Therefore, co-cultivating microalgae with fungi or activated sludge have been identified as efficient systems for wastewater treatment in addition to yield valuable products from biomass. However, for fungal–microalgal or bacterial–microalgal interactions, the influencing factors such as detailed mechanisms, optimization of suitable strains, cultivation condition, light intensity, light photoperiod, etc. are still unclear, and need further, deep research. In fact, not all filamentous fungal strains or all bacteria can form pellets and induce prominent role in biogas upgrading and simultaneous biogas slurry decontamination during microalgae co-cultivation operation.

Light wavelength and intensity are essential parameters for microalgae growth and photosynthesis, which are connected with the microalgae carbon fixation [7]. However, natural light is not stable for microalgae growth because the light intensity is low on rainy days and excessive at noontime on sunny days, especially in summer, which may consequently cause insufficient light irritation and photoinhibition. As an artificial light source, light-emitting diode (LED) has specific narrow bands which can produce cost effective irradiance, acting as the optimum spectral for growth of typical microalgae strains [21]. Cheirsilp and Torpee [22] revealed that higher efficiency and economical maintenance of microalgal photosynthesis systems were achieved using specific narrow band light frequencies, such as LED sources, comparing with the use of natural light sources which may not contain the absorption frequencies required for chlorophyll. Meanwhile, a series of experiments has been carried out by Yan et al. [2,23,24,25,26] and an incremental light intensity strategy was established for efficient and economical biogas upgrading and simultaneous biogas slurry nutrient removal by mono-cultivation of selected microalgae strain. However, systematic study about the effects of LED light wavelength, light intensity strategy and light photoperiod on simultaneous biogas upgrading and biogas slurry nutrient reduction by different microalgal-based cultivation approaches such as algal–algal, algal–fungal and algal–bacterial remains largely unknown.

Aiming at these unsolved problems, this research focused on nutrient and CO_2_ uptake by different microalgal-based cultivation approaches, i.e., mono-cultivation of microalgae, co-cultivation of microalgae with fungi, and co-cultivation of microalgae with activated sludge under various light intensities and photoperiods in photobioreactors. The lighting control strategy and appropriate cultivation technology were optimized by analyzing the biomass reproduction, as well as the removal efficiencies of CO_2_, COD, TN and TP under various light intensities and photoperiods using three microalgal-based cultivation approaches, i.e., algal, algal–fungal and algal–bacterial cultivation systems.

## 2. Methods and Materials

### 2.1. Collection of Algal Strains and Culturing Conditions for the Selected Microalgal-Based Technologies

#### 2.1.1. Culture 1: Mono-Cultivation of Microalgae Strain

*Chlorella vulgaris* (*C. vulgaris*, FACHB-31) was selected based on its high biogas tolerance and fast specific growth rate in high nutrient concentration wastewater [20,25]. Then, they were cultured on BG-11 medium after being autoclaved. The BG-11 medium contained NaNO_3_ (1500 mg L^−1^), K_2_HPO_4_·3H_2_O (40 mg L^−1^), MgSO_4_·7H_2_O (75 mg L^−1^), CaCl_2_·2H_2_O (36 mg L^−1^), ferric ammonium citrate citric acid·H_2_O (6 mg L^−1^), EDTA-Na_2_ (1 mg L^−1^), Na_2_CO_3_ (20 mg L^−1^), and A_5_ (1 mg L^−1^). The content of A_5_ consisted of H_3_BO_3_ (2860 mg L^−1^), MnCl_2_·H_2_O (1860 mg L^−1^), ZnSO_4_·7H_2_O (222 mg L^−1^), CuSO_4_·5H_2_O (79 mg L^−1^), NaMoO_4_·2H_2_O (390 mg L^−1^), and CoCl_2_·6H_2_O (49 mg L^−1^) [3]. These microalgal strains were cultivated in 500 mL Erlenmeyer flasks for 7 days to proliferate for further experiments. The detailed cultivation conditions were as follows: cool-white light with a light intensity of 200 μmol m^−2^ s^−1^, photoperiod of light 12 h:dark 12 h, temperature at 25 ± 0.5 °C, 7 days of experimental duration time and artificial oscillation three times a day (8:00 a.m., 2:00 p.m., and 8:00 p.m.) at speed of approximately 200 rpm. Such mono-cultivation was carried out in an illuminating incubator (GZP-350S, Shanghai Jing Hong Laboratory Instrument Co., Ltd., Shanghai, China). LED lamps were installed on three sides of the incubators. The dry weight (DW) of selected microalgal strains in the stock culture was nearly 135.28 ± 12.09 mg·L^−1^.

#### 2.1.2. Culture 2: Co-Cultivation of Microalgae with Fungi

*Ganoderma lucidum* (*G. lucidum*, 5.765) was obtained from China General Microbiological Culture Collection Center and selected for this experiment because of its high specific growth rate and high pelletization performance with *C. vulgaris* based on our preliminary studies. For pelletization, every 5 mL spore solution was cultivated at 25 ± 0.5 °C for 7 days in 100 mL synthetic growth medium (glucose, 10 g L^−1^; NH_4_NO_3_, 2.0 g L^−1^; K_2_HPO_4_, 1.0 g L^−1^; NaH_2_PO_4_·H_2_O, 0.4 g L^−1^; MgSO_4_·7H_2_O, 0.5 g L^−1^; and yeast extract, 2.0 g L^−1^; pH 6.5). Afterwards, algal cultures (100 mL) were centrifuged, washed and resuspended to achieve a final total suspended solid (TSS) concentration of about 153.45 ± 13.77 mg L^−1^, and then mixed with *G. lucidum* suspension (5 mL, about 84 ± 7 mg L^−1^ DW) for further co-cultivation in the photobioreactors. The algal–fungal mixtures were shaken at 160 rpm for 48 hat constant light intensity of 200 μmol m^−2^ s^−1^, light-dark cycle of 12 h:12 h, and temperature of 25 ± 0.5 °C [3]. The DW of cultured microalgal–fungal pellets was about 136.52 ± 9.67 mg L^−1^.

#### 2.1.3. Culture 3: Co-Cultivation of Microalgae with Activated Sludge

The 200 mL of nitrifying-denitrifying activated sludge (8.56 g TSS L^−1^) was derived from a wastewater treatment plant of Shanghai and mixed with 1 L cultured microalgae broth. The photoperiod, light intensity and temperature are the same as Culture 2. The initial TSS concentration in the co-cultivation broth of the biogas slurry was about 135.51 ± 9.06 mg L^−1^.

### 2.2. Biogas Slurry and Biogas

Crude biogas and biogas slurry were obtained from a farm biogas plant in Jiayuan green meadow. Firstly, raw biogas was desulfurized by chemical absorption reactors until the H_2_S concentration was inferior to 100 ppm. Afterwards, the components of crude biogas were measured as 64.59 ± 3.27% (*v*/*v*) CH_4_, 33.79 ± 1.56% (*v*/*v*) CO_2_, 0.38 ± 0.03% (*v*/*v*) O_2,_ and 1.23 ± 0.11% (*v*/*v*) H_2_O. Raw biogas slurry was pretreated by passing through a glass microfiber filter (GF/C; Shanghai Bestest Biological Technology Co., Ltd., Shanghai, China) and ultraviolet sterilized (KCJ-10W; Konche (Shenzhen) Water Treatment Co., Ltd., Shenzhen, China) to avoid potential interference from impurities sediment and microorganisms. The characteristics of biogas slurry before and after pretreatment are listed in Table 1. 

### 2.3. Photobioreactor

The photobioreactor comprises two interconnected 16.8 L (individual) glass-made cylinder blocks (total height = 0.6 m; diameter = 0.2 m) filled with 14 L raw biogas and 2.8 L biogas slurry. For details on the experimental device, refer to our previously published work [3]. The experimental time was 10 days.

### 2.4. Experimental Procedure

Batch experiments were carried out to remove nutrient in biogas slurry and CO_2_ in biogas using microalgal mono-cultivation and co-cultivation. Firstly, three photoperiods (i.e., 12 h light:12 h dark cycle, 14 h light:10 h dark cycle and 16 h light:8 h dark cycle) were settled for each culture (i.e., Culture 1, Culture 2 and Culture 3). Other constant parameters of cultivation conditions were as follows: cool-white light with light intensity of 200 μmol m^−2^ s^−1^, temperature at 25 ± 0.5 °C and 10 days of experimental time. Secondly, the two relatively optimal co-cultivation methods were selected based on the specific growth rate as well as nutrient and CO_2_ removal efficiency. The different light intensity (250, 450, and 650 μmol m^−2^ s^−1^) was screened, and illuminated with the optimal photoperiod which was determined as the optimal photoperiod in the first step. During 10 days of the operation time, the experimental groups were periodically sampled for evaluation of biomass grow rates, mean daily productivity, biogas purification and the biogas slurry nutrients removal efficiency. Lastly, both the light intensity and photoperiod strategy were optimized by analyzing the economic efficiencies of the biogas CO_2_ upgrading and the biogas slurry nutrient removal.

### 2.5. Sampling and Analyses

The biogas was sampled from the port of the photobioreactor for components analysis (CH_4_, CO_2_, O_2_ and H_2_O) using a circulating gas analyzer (GA94; ONUEE Co. Ltd., Shenzhen, China). Each 50 mL co-cultivated suspensions were filtered by a glass microfiber filter (GF/C, Whatman, Boston, MA, USA), and dried at 100 °C for 24 h before being cooled to room temperature in a desiccator. The DW of the microalgae was calculated as the difference between the filter weight before and after filtration. The filtrates were analyzed for COD, TN, and TP according to a standard method [27]. All experiments were run in triplicate, and the results were averaged. 

Special growth rate (*μ*, d^−1^), mean daily productivity (*P*, g L^−1^ d^−1^), biogas CO_2_ and total biogas slurry nutrient removal efficiency (RE, %) and the economic efficiency of the biogas CO_2_ or biogas slurry nutrient removal (*E*, USD^−1^) were experimentally estimated by following Equations (1)–(4) [3,11,20].

(1) $$\mu =\frac{\ln C_{i}-\ln C_{0}}{t}$$

(2) $$P=\frac{DW_{i}-DW_{0}}{\left(t_{i}-t_{0}\right)d}$$

(3) $$\mathrm{RE}=\left(1-\frac{C_{i}}{C_{0}}\right)\times 100$$

(4) $$E=\frac{\mathrm{RE}}{kTp}$$

where *DW_i_* and *DW*_0_ are the biomass DW (g L^−1^) at time *t_i_* and *t*_0_ (initial time), *d* is experimental duration time (days), *C_i_* and *C*_0_ are biogas CO_2_ content (%, *v*/*v*) or biogas slurry nutrient concentration (g L^−1^) at time *t_i_* and *t*_0_, k is the electric power charge per unit of power consumption (USD kW^−1^ h^−1^), *T* is the illumination time (h), and *p* is the LED electrical power consumption (*W*). Normally, the electric power charge per unit of power consumption k in local is around 0.645 RMB kW^−1^ h^−1^, equivalent to 0.096 USD kW^−1^ h^−1^, based on the price level in Jiaxing City, China.

### 2.6. Statistical Analyses

All statistical analyses in this study were carried out via SPSS software (Version 19.0, Statistical Product and Service Solutions China, Shanghai, China). One-way analysis of variance was used to determine whether there was a significant difference between the variables, such as light intensity, photoperiod, and microalgal-based cultivation approaches. Duncan’s multiple range tests was used to analyze the difference between groups. The threshold for significance was set at *p* ≤ 0.05. 

## 3. Results and Discussion

### 3.1. The Strains Growth for the Selected Three Microalgae-Based Technologies at Different Photoperiod Treatments

Specific growth rates and mean daily productivity in these cultures were measured under different photoperiods as 12 h light:12 h dark cycle (short), 14 h light:10 h dark cycle (middle) and 16 h light:8 h dark cycle (long), and the results shown in Table 2. Variation trends of specific growth rates as well as mean daily productivity of all culture methods were similar. That is, a high specific growth rate was achieved under middle photoperiod (14 h light:10 h dark cycle), followed by short photoperiod (12 h light:12 h dark cycle), and the lowest specific growth rates was observed under long photoperiod (16 h light:8 h dark cycle). Both specific growth rate and mean daily productivity in Culture 2 were higher than those in Culture 1 and Culture 3. 

There are three probable reasons for these special experimental phenomena. Firstly, light was the only energy source in photoautotrophic conditions in this study. Furthermore, sufficient light can enhance the growth of photoautotrophic biomass significantly, as the light photons are absorbed by biomass as their nutrients [21,28]. Nevertheless, a high probability to cause photo inhibition was already observed by striking the light harvesting complex of cells at its peak electrical energy due to its shorter wavelength, high light intensities or excessive/limited lighting strategy [29]. This finding was consistent with the conclusions reported by Yan et al. [2], who studied the microalgal DW under various lighting scenarios of light intensities and photoperiods during 7 days and the highest value was achieved under middle light photoperiod. In conclusion, the optimal light photoperiod was 14 h light:10 h dark cycle in this study, because short/long photoperiod (12 h light:12 h dark cycle or 16 h light:8 h dark cycle) may support limited/excessive light illumination which can induce photo inhibition for biomass reproduction. 

Secondly, microbial cells in these three cultures have different abilities for metabolism and photosynthesis based on CO_2_ sequestration in the bioreactor, which was the key mechanism for biomass reproduction [2]. Therefore, microalgal–fungal and microalgal–bacterial systems have greater ability for taking up CO_2_ through photosynthesis than mono-cultivation [3]. Besides, the metabolic process of these microbial cells can significantly produce ATP by assimilating the nutrients in biogas slurry, which was utilized in return as the enzyme activator during the photosynthetic CO_2_ uptake process [14]. Such CO_2_ uptake and nutrients adsorption by these microalgal–fungal and microalgal–bacterial cells played a key role in microalgae cell growth [2]. In detail, for co-cultivation with microalgae and fungi, pelletization was usually found by microalgae and filamentous fungi, which can greatly enhance biomass reproduction [30,31]. Nowadays, it is well-known that coagulative and non-coagulative effects contributed much to pelletization progress, and then induced high specific growth rate and mean daily productivity. The coagulative mechanism includes spore coagulation resulting in the developments of aggregates/pellets, while the non-coagulative mechanism facilitates germination of the spores into hyphae and intertwinement into the pellets [30,32]. Furthermore, another probable reason was reported by Muradov et al. [30], who revealed that the negative surface charge of algal cells (−23.7 mV) can potentially be neutralized when exposed to fungal hyphae and mycelia, which are positively charged (+46.1 mV), therefore enabling attachment of algal cells to fungal cell walls, improving productivity of the resulting biomass. Besides, for co-cultivation of microalgae and bacteria, specific growth rate and mean daily productivity is higher than that in Culture 1. That is, microalgal growth could also increase by microalgae growth-promoting bacteria (MGPB), which was consistent with some previous studies [33,34,35]. Specifically, De-Bashan et al. [34] have conducted series study on a novel approach for removing ammonium and phosphorus from municipal wastewater by MGPB and revealed that significant improvement of xenobiotics has been observed during microalgal–bacterial co-culture/co-immobilization. In addition, increased and improved cellular metabolism, cell density and cell size of microalgae induced by MGPB evidenced in chlorophyll pigment, cell size, cell cytology, lipid content, microalgal population size and variety of fatty acids had been reported, which resulted in enhanced removal of ammonium and phosphate from wastewater. Similarly, a cooperative microalgal–bacterial system has already been developed by us to remove H_2_S and CO_2_ from biogas by three microalgal strains (*C. vulgaris*, *S. obliquus*, and *N. oleoabundans*) mixed with activated sludge using biogas slurry as nutrient medium [20]. In conclusion, both bacteria and fungi can increase the efficiency of bio-flocculation of microalgae, which would significantly induce high biomass reproduction, under appropriate light intensity and photoperiod.

Thirdly, as is well-known, the pH is an important factor for cultivation condition in the photobioreactor. Normally, common problems associated with culture media are the use of an inadequate pH and high levels of precipitate resulting from incorrectly formulated media, including omission of vital ingredients [36]. Recently, in view of great importance of pH effect on pellet formation, pH adjustment was used to induce the formation of fungal cell pelletization, providing a simplified method by which to facilitate the oleaginous cell harvest [19,31]. Especially, Rachlin and Grosso [36] studied the effects of pH on the growth response of the green alga *C. vulgaris* and revealed that optimal growth of the microalgae occurred when the pH of the medium was adjusted to values of 6.9–8.0, while acidic (3.0–6.2) and alkaline (8.3–9.0) pH values retarded the growth of this alga. In this study, variations of pH under photoperiod with the three cultures are shown in Table 3. Obviously, varied from 6.81 to 7.21, pH was time-dependent and rise slightly with experimental time. However, no significant difference was found among the three cultures under each photoperiod in the same cultivation time (*p* > 0.05). Consequently, it is of great importance for pH to affect strains growth in the cultivation for biogas upgrading and simultaneously biogas slurry nutrients removal, but all the variation of pH remains in the optimal domain for biomass reproduction under all the culture condition in this operation time. Therefore, pH was not the upmost important factor that affects the microalgal growth and mean daily productivity in this study. 

### 3.2. Nutrient Removal Efficiencies at Different Photoperiod Treatments

**Figure 1:**
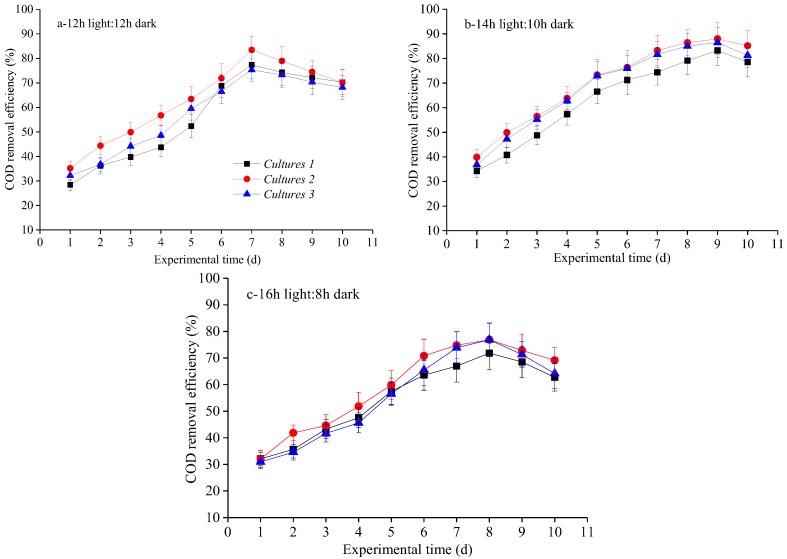
COD removal efficiency over time under various light photoperiods for three cultivation approaches: (**a**) 12 h light:12 h dark; (**b**) 14 h light:10 h dark; and (**c**) 16 h light:8 h dark.

The COD% removal efficiencies of the three cultures were evaluated at different photoperiods, and the results are displayed in Table 4 and Figure 1. Based on Table 4, the highest mean COD% removal efficiency was achieved in Culture 2, which was 2.5% and 9% higher than the highest mean COD% removal efficiency in Culture 1 and Culture 3, respectively. This result indicated that the microalgal specific growth rate match COD% removal efficiency. In the experiment, all the three microalgal-based cultivation approaches obtained optimal COD% removal efficiency at 14 h light:10 h dark cycle, further supporting this light photoperiod as being optimal for biogas slurry nutrient removal (Table 4). Average and maximum COD% removal efficiency in Culture 2 at 14 h light:10 h dark cycle showed the highest, although a little higher than those achieved in Culture 3. Anyway, co-cultivation of microalgae with fungi in Culture 2 seems to be the optimum for biogas slurry COD removal among the three microalgae-based treatment systems. These results are comparable with previously reported literature, where Wang et al. [10] reported that 68.11% and 64.67% of COD was removed by *N. palea* and *C. vulgaris* during the moderate photoperiod averagely. Similarly, Yan and Zheng (2013) reported that *Chlorella* sp. can remove 36–86% (average 52%) of COD from biogas slurry within 24 h under short (12 h light:12 h dark), moderate (14 h light:10 h dark), and long (16 h light:8 h dark) photoperiods [26]. Zhao et al. [11] also indicated that the maximum COD removal from biogas slurry can reach approximately 85.35%. Variations in productivities established between different studies probably result from different light intensity, different photoperiod, species of microalgae utilized, temperature, source and proportion of carbon dioxide applied, as well as the type of photo-bioreactor system applied in the experiment. In addition, the highest COD% removal efficiency was obtained in 0–7 days, 0–9 days and 0–8 days under short, middle and long photoperiods based on the Figure 1, respectively. This result further confirmed that slightly prolonged duration time by microalgal–fungal and microalgal–bacterial mixture cultivated in Culture 2 and Culture 3 attributed much to COD% removal efficiency in the biogas slurry under certain photoperiod. Reproduction process may slow down due to the nutrient depletion and toxic metabolic product accumulation during the reproduction processes, which subsequently induce declining of COD% removal efficiency in the last three days for each photoperiod in this study. This was coincident with the results of Zwietering et al. [37], who reported that biomass reproduction process slowed down when death rate was greater or equal to birth rate of microalgae cells. Lastly, carbon assimilation into biomass is the main carbon removal mechanism based on carbon mass balance over the entire experimental period [38]. Microalgal, microalgal–fungal and microalgal–bacterial strains cultivated independently in this study can sustain both heterotrophic and autotrophic growth with CO_2_ as the sole carbon source and induced COD removal in the biogas slurry subsequently. The carbon existed in the biogas and biogas slurry accounts for approximately 50% of microalgal biomass and acts as fundamental element in microalgal cells [20]. To sum up, these results indicate that the appropriate selection of microalgae-based cultivation approaches and light photoperiod are effective operational strategy to increase COD% removal efficiency.

**Figure 2:**
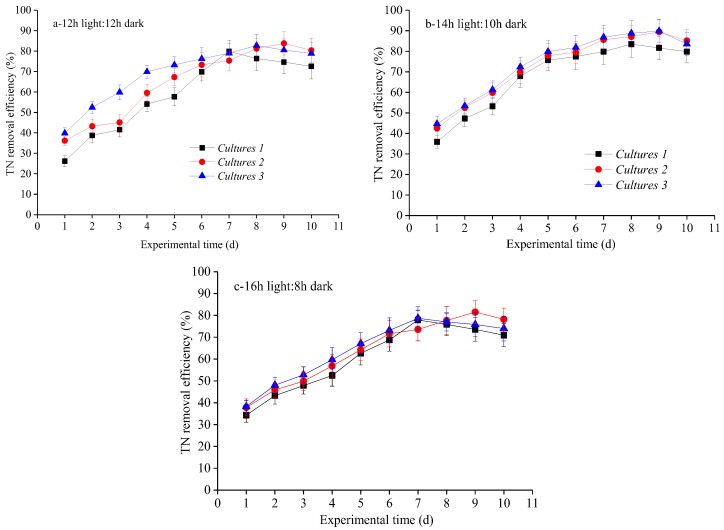
TN removal efficiency over time under various light photoperiods for three cultivation approaches: (**a**) 12 h light:12 h dark; (**b**) 14 h light:10 h dark; and (**c**) 16 h light:8 h dark.

Besides, TN in biogas slurry was also removed significantly in the three cultures separately under the three photoperiods (Table 4 and Figure 2). However, the removal efficiencies of these strains differed significantly during 10 days. Under the optimal photoperiod of 14 h light:10 h dark cycle, the highest average TN% removal efficiency was achieved in Culture 3, followed by Culture 2 and Culture 1. This result indicated that the TN removal is not correlated with microalgal specific growth rate, neither to COD% removal efficiency. The possible reason for that is addition of nitrifying–denitrifying activated sludge as bacteria in Cultures 3, which induced relatively high TN% removal efficiency under middle light photoperiod. In this study, all the optimal average TN% removal efficiency in the three cultures was also obtained under 16 h light:8 h dark cycle (Table 4). When concerned with long photoperiod, average and maximum TN% removal efficiency were observed in Culture 3, which was higher than the highest TN% removal efficiency achieved in Culture 2 and Culture 1. Therefore, co-cultivation of microalgae with bacteria as activated sludge in Culture 2 seems to be the optimal procedure for TN removal in the biogas slurry, and optimal light photoperiod was 14 h light:10 h dark cycle. The TN was mainly reduced by assimilating microbial photosynthesis because microalgal, microalgal–fungal and microalgal–bacterial reproduction in the three cultures require abundant nitrogen to build nucleic acids and proteins [39]. Hence, assimilation into biomass was the principal mechanism of N removal in the study. This finding is consistent with previous reported results [3,17,18,19]. For instance, Yan et al. [2] studied the effects of various light wavelengths, light intensities and photoperiods on biogas upgrading and simultaneously biogas slurry decontamination and revealed that 74.53% of TN was removed. Zhao et al. [40] also indicated that the average TN removal from biogas slurry can reach approximately 45.73–62.51% according to different initial CO_2_ concentration. Our previous work revealed that enhanced average TN% removal efficiency was observed as 61.12–73.24% by co-cultivation of *C. vulgaris* and *G. lucidum* [3]. 

**Figure 3:**
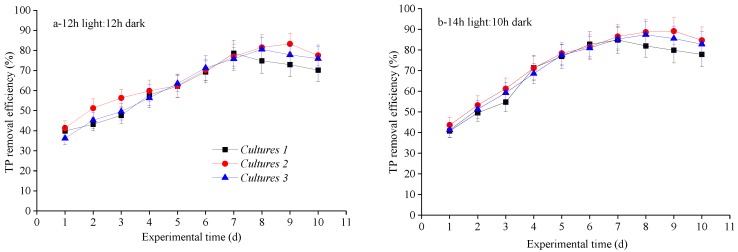

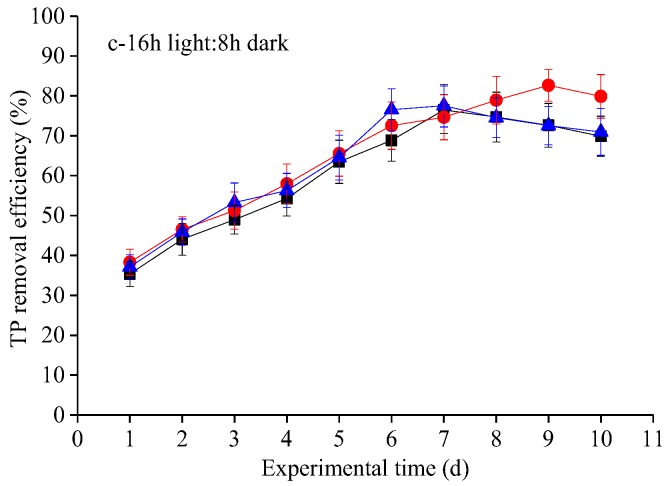
TP removal efficiency over time under various light photoperiods for three cultivation approaches: (**a**) 12 h light:12 h dark, (**b**) 14 h light:10 h dark; and (**c**) 16 h light:8 h dark.

As shown in Table 4, cultivation approaches, light photoperiods, and the combined effect of these parameters had significant effects on TP% removal efficiency. The variation trends of TP% removal efficiency (Figure 3) agreed with those of COD% removal efficiency and biomass reproduction (Figure 1, Table 2), but were not the same as TN% removal efficiency (Figure 2). Phosphorus is an important nutrient in algal production as a constituent of phospholipids for cell membranes and of adenosine triphosphate to supply energy for cell functions, despite assimilation into biomass [10]. More in detail, average and maximum TP% removal efficiency were observed in Culture 2, a little higher than those achieved in Culture 3 and Culture 1. Furthermore, all three cultures obtained optimal average TP% removal efficiency at 14 h light:10 h dark photoperiod (Table 4). Therefore, similar to TN% removal efficiency, Culture 2 with 14 h light:10 h dark cycle also seems to be the optimum for TP removal in the biogas slurry. These findings are similar to those reported by Yan et al. [24,25], who reported maximal TP removal efficiencies was 67.54–82.06%. Similarly, our previous work focused on optimization for different cultivation approaches, and the maximum TP% removal efficiency was 76.69 ± 6.97% by co-cultivation of *C. vulgaris* and *G. lucidum* [3]. Therefore, the nutrients in the biogas slurry were efficiently decontaminated during the biogas upgrade process with biomass reproduction. 

To sum up, the results of microalgae DW growth partly agreed with biogas slurry nutrients removal. Specifically, the biogas slurry nutrients (i.e., COD, TN, and TP) were reduced efficiently by Culture 1, Culture 2 and Culture 3 under the photoperiod treatments. Culture 2 seems to be the optimal procedure for COD, TN and TP removal efficiency in the biogas slurry under 14 h light:10 h dark cycle. Both microalgal–fungal and microalgal–bacterial co-cultivation systems are superior to mono-cultivation approaches for biogas slurry nutrients removals under 14 h light:10 h dark cycle.

### 3.3. Biogas Upgrading

**Figure 4:**
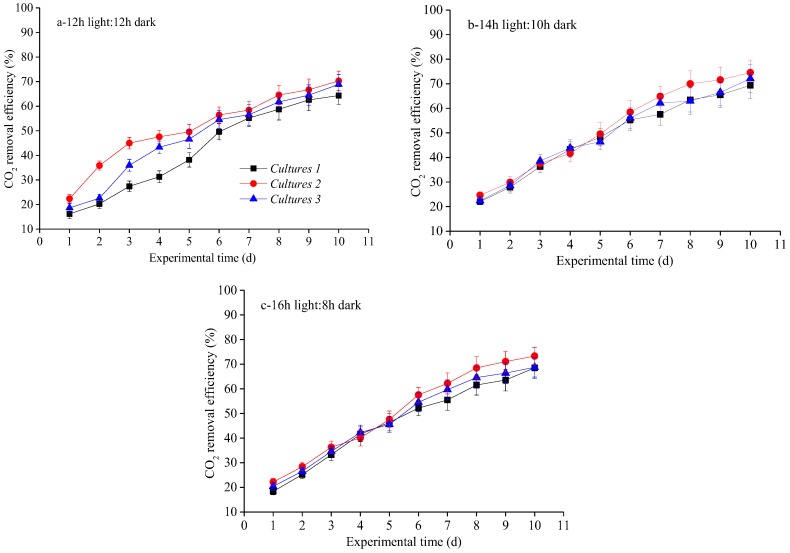
CO_2_ removal efficiency over time under various light photoperiods for three cultivation approaches: (**a**) 12 h light:12 h dark; (**b**) 14 h light:10 h dark; and (**c**) 16 h light:8 h dark.

CO_2_ is the vital carbon source of microalgal, fungal and/or bacterial cells in this experiment: approximately half of the microbial cells DW can be attributed to CO_2_-derived carbon [3]. Therefore, the biogas components (i.e., CO_2_, CH_4_, H_2_O and O_2_) were investigated over time, using three different microalgal-based cultivation approaches under three different photoperiods. During the experimental operation time, CO_2_% removal efficiency increased significantly with all microalgal, microalgal–fungal and microalgal–bacterial cultivation technologies under photoperiods (Figure 4). Furthermore, Culture 2 and Culture 3 systems seem more efficient for CO_2_ removal than Culture 1. Specifically, for all the three cultures, the highest CO_2_ removal efficiency was obtained under middle photoperiod (14 h light:10 h dark cycle), followed by the long photoperiod (16 h light:8 h dark cycle) and short photoperiod (12 h light:12 h dark cycle). With Culture 2, for example, average and maximum of CO_2_ remove efficiency achieved 52.26 ± 5.37% and 74.54 ± 5.13%, respectively, at the end of the operation time under middle photoperiod, followed by 50.68 ± 4.75% and 73.28 ± 3.47% with long photoperiod, and finally 51.66 ± 4.65% and 70.28 ± 3.97% with short photoperiod, which were in agreement with results from Zhao et al. [11], who reported maximum biogas CO_2_ removal efficiencies of 81.68 ± 3.28% at low light intensity with long photoperiods, 86.15 ± 3.94% at moderate light intensity with middle photoperiods and 53.25 ± 3.21% at high light intensity with short photoperiods. In the present study, the efficiency of biogas upgrading in different cultivation systems agreed with trends observed for specific growth rate (d^−1^) and mean daily productivity (g L^−1^ d^−1^) (Table 2), partly because nearly half of the whole biomass reproduction was made up of CO_2_-derived carbon. Moreover, CO_2_ was mainly consumed through microalgal photosynthesis in the biogas slurry. The medium was found to have significant effect on CH_4_ content (*v*/*v*) enrichment in upgraded biogas, irrespective of the microalgal strains present, which further confirm the prominent role of co-cultivation with fungi or bacteria [12]. This result agrees with the findings of previous studies by Sun et al. [20], who reported that CO_2_ can be reduced by 49.95–62.31% by microalgal–bacterial co-cultivation. Similarly, Zhang et al. [3] also reported that CO_2_% removal efficiency using microalgal–activated sludge ranged between 66.93% and 88.27%, which is higher than using mono-cultivated microalgae (54.79–74.65%) or co-cultivated microalgae and fungi (56.15–83.31%). In this study, the methane content was enriched significantly because the CO_2_ content in the biogas was effectively reduced by the photosynthesis of microalgae when co-cultivated with fungi or activated sludge in the photobioreactor with middle photoperiod (14 h light:10 h dark cycle). Hence, CO_2_ removal during the biogas upgrading provides an additional strategy for GHG emission control by reincorporation it into the biogas production process. 

### 3.4. Economic Efficiency of the Energy Consumption

Using three different microalgal-based cultivation approaches, the economic efficiency of the energy consumption for both biogas and nutrient removal under various photoperiods were evaluated according to the calculation of Equation (4), and the results are shown in Table 4. The results for CO_2_, COD, TN, and TP removal efficiencies had the same trend. The Economic efficiency of the energy consumption for COD% removal efficiency, TN% removal efficiency, TP% removal efficiency and CO_2_% removal efficiency by co-cultivated microalgae–fungi (Cultures 2) was significantly higher than other two cultures. These findings agreed with the results of microalgae specific growth rate, mean daily productivity, removal efficiency of CO_2_ in biogas and biogas slurry nutrient. As a result, the optimal cultivation approach for removal of CO_2_ in biogas according to economic efficiency was co-cultivation of *C. vulgaris* with *G. lucidum* under photoperiod of 14 h light:10 h dark cycle.

### 3.5. Optimization of LED Light Intensities Treatments for the Selected Two Cultures

According to above-mentioned conclusions, Culture 2 and Culture 3 have significant effects on biogas upgrading and biogas slurry nutrients removal under optimal photoperiods as 14 h light:10 h dark cycle during 10-day operation schedule. However, they were obtained under stationary light intensity of 250 μmol m^−2^ s^−1^. Therefore, it was still unclear whether such selected light intensity was the most optimal one for microalgal–fungal and microalgal–bacterial system. Aiming at such unsolved problems, the effect of light intensity on nutrient and CO_2_ removal for the two co-cultivation approaches under the optimal photoperiods (14 h light:10 h dark) were evaluated and the results are displayed in Table 5. 

Based on Table 5, both co-cultivation (Culture 2 and Culture 3) under light intensity of 250–650 μmol m^−2^ s^−1^ achieved higher mean daily productivity, CO_2_ removal efficiency and biogas slurry nutrient removal efficiency than those under light intensity of 200 μmol m^−2^ s^−1^ (Table 4 and Table 5). Furthermore, under the optimal light intensity of 450 μmol m^−2^ s^−1^, the mean daily productivity, together with biogas slurry nutrients removal efficiency for COD, TN and TP in Culture 2, was the highest. Similar variation trend was found in Culture 3. Surprisingly, the highest CO_2_ removal efficiency was observed under low light intensity at 250 μmol m^−2^ s^−1^ in both cultures, followed by the moderate and high light intensity. Therefore, both microalgal–fungal and microalgal–bacterial systems have remarkable effects on biogas upgrading and simultaneously biogas slurry nutrients removal because no significant difference was found between these methods (*p* > 0.5). In short, the optimal light intensity for nutrients and CO_2_ removal were 450 μmol m^−2^ s^−1^ and 250 μmol m^−2^ s^−1^, respectively. Ouyang et al. [41] investigated the ability of microalgae to remove biogas slurry nutrients and to upgrade biogas simultaneously under various LED light intensities and revealed that 150–170 μmol m^−2^ s^−1^ was the most suitable light intensity. Similarly, Ho et al. [42] reported that a light intensity of 220–240 μmol m^−2^ s^−1^ is optimal for microalgal growth (indigenous *S. obliquus*). This inconsistency between our results and other research might be attributed to the different species, different photoperiod or different cultivation condition applied in its corresponding experiments. 

Generally, adequate illumination is the most important factor affecting microalgal, fungal, and bacterial growth. Thus, an increase in the light intensity can enhance biomass reproduction of the microbial cells at a certain range. However, once the light intensity exceeds the light saturation limit level, the microalgae would be damaged by excessive light energy because high light intensities may lead to growth inhibition, photosystems overload, photosystem damage, photo oxidation and/or photo inhibition [21]. Inversely, if the light intensity is too low to maintain the ordinary microalgae growth, defective biomass reproduction would occur and as a result induce a declining effect for biogas upgrading and biogas slurry decontamination. Therefore, these findings suggest that controlling the light intensity is a vital operating procedure to manipulate microalgal, microalgal–fungal and microalgal–bacterial growth. For moderate light intensity, 450 μmol m^−2^ s^−1^ in this study, during 10 days, the microalgal–fungal and microalgal–bacterial cells reproduced rapidly, possiblu because the nutrients in the biogas slurry culture were sufficient and the metabolic waste had not accumulated thickly. Anyway, the nutrient in the biogas slurry was efficiently reduced during the biogas upgrading process under moderate light intensity of 450 μmol m^−2^ s^−1^.

## 4. Conclusions

Co-cultivation of *C. vulgaris* with *G. lucidum* or activated sludge promoted the biogas upgrading and biogas slurry nutrient removal under various light intensities and photoperiods in photobioreactors. Removing nutrient and CO_2_ with Co-culturing of *C. vulgaris* and *G. lucidum* under the photoperiod of 14 h light:10 h dark and moderate light intensity of 450 μmol m^−2^ s^−1^ were regarded as the optimal strategy. More than 70% of mean COD, TN and TP in addition to more than 52% of mean CO_2_ were removed. The analyses of economic efficiency also showed the economic superiority of the selected strategy for biogas slurry purification and biogas upgrading in this study. 

## Figures and Tables

**Table 1:** The characteristics of biogas slurry in this study.

Parameter	Before Pretreatment	After Pretreatment
pH	6.78 ± 0.18	6.97 ± 0.17
Dissolved oxygen (mg L^−1^)	5.67 ± 0.44	5.43 ± 0.39
Dissolved inorganic carbon (mg L^−1^)	982.14 ± 32.73	971.08 ± 41.39
COD (mg L^−1^)	1521.75 ± 47.19	1495.62 ± 59.94
TN (mg L^−1^)	289.78 ± 29.37	278.46 ± 30.13
TP (mg L^−1^)	29.82 ± 3.26	27.98 ± 2.87

**Table 2:** Specific growth rates and mean daily productivity of mono-cultivation of microalgae (Culture 1), co-cultivation of microalgae with fungi (Culture 2) and co-cultivation of microalgae with activated sludge (Culture 3) under different photoperiods.

	Culture 1	Culture 2	Culture 3
Specific Growth Rate (d^−1^)			
12 h light:12 h dark	0.279 ± 0.07	0.362 ± 0.07	0.325 ± 0.06
14 h light:10 h dark	0.308 ± 0.08	0.381 ± 0.09	0.337 ± 0.07
16 h light:8 h dark	0.257 ± 0.05	0.353 ± 0.08	0.296 ± 0.05
Mean daily productivity (g L^−1^ d^−1^)			
12 h light:12 h dark	0.085 ± 0.007	0.143 ± 0.011	0.129 ± 0.011
14 h light:10 h dark	0.098 ± 0.006	0.162 ± 0.013	0.147 ± 0.010
16 h light:8 h dark	0.081 ± 0.007	0.134 ± 0.012	0.113 ± 0.009

**Table 3:** Variations in pH under various photoperiods for three cultivation approaches.

Cultivation Approaches/Photoperiods	Time (h)										
	0	24	48	72	96	120	144	168	192	216	240
Culture 1											
12 h light:12 h dark	6.87 ± 0.16	6.91 ± 0.21	6.94 ± 0.18	6.98 ± 0.26	7.03 ± 0.24	7.05 ± 0.31	7.07 ± 0.33	7.09 ± 0.29	7.13 ± 0.29	7.16 ± 0.34	7.19 ± 0.34
14 h light:10 h dark	6.83 ± 0.14	6.93 ± 0.27	6.96 ± 0.19	7.01 ± 0.21	7.05 ± 0.25	7.07 ± 0.24	7.13 ± 0.34	7.16 ± 0.21	7.19 ± 0.31	7.21 ± 0.36	7.17 ± 0.29
16 h light:8 h dark	6.81 ± 0.19	6.92 ± 0.23	6.97 ± 0.22	7.05 ± 0.29	7.08 ± 0.27	7.06 ± 0.27	7.14 ± 0.35	7.18 ± 0.25	7.21 ± 0.32	7.15 ± 0.27	7.19 ± 0.41
Culture 2											
12 h light:12 h dark	6.79 ± 0.17	6.82 ± 0.21	6.86 ± 0.25	6.92 ± 0.27	7.06 ± 0.22	7.08 ± 0.31	7.13 ± 0.24	7.15 ± 0.32	7.17 ± 0.33	7.14 ± 0.21	7.16 ± 0.33
14 h light:10 h dark	6.83 ± 0.12	6.87 ± 0.22	6.91 ± 0.19	6.97 ± 0.23	7.07 ± 0.29	7.09 ± 0.33	7.14 ± 0.28	7.16 ± 0.31	7.19 ± 0.31	7.11 ± 0.23	7.15 ± 0.35
16 h light:8 h dark	6.86 ± 0.15	6.91 ± 0.29	6.94 ± 0.26	6.99 ± 0.24	7.02 ± 0.21	7.05 ± 0.29	7.08 ± 0.23	7.12 ± 0.34	7.15 ± 0.37	7.19 ± 0.34	7.17 ± 0.37
Culture 3											
12 h light:12 h dark	6.77 ± 0.23	6.83 ± 0.24	6.87 ± 0.21	6.93 ± 0.21	7.05 ± 0.21	7.08 ± 0.32	7.12 ± 0.35	7.13 ± 0.32	7.14 ± 0.37	7.18 ± 0.38	7.14 ± 0.21
14 h light:10 h dark	6.85 ± 0.15	6.88 ± 0.21	6.92 ± 0.23	6.98 ± 0.25	7.01 ± 0.23	7.04 ± 0.23	7.08 ± 0.31	7.11 ± 0.36	7.13 ± 0.32	7.11 ± 0.35	7.16 ± 0.32
16 h light:8 h dark	6.82 ± 0.18	6.86 ± 0.19	6.89 ± 0.22	6.95 ± 0.22	7.03 ± 0.28	7.09 ± 0.29	7.12 ± 0.27	7.17 ± 0.34	7.19 ± 0.35	7.13 ± 0.31	7.18 ± 0.34

**Table 4:** Mean values ± SD of the removal efficiency and the economic efficiency of biogas CO_2_ and biogas slurry nutrient removal under different photoperiods for three cultivation approaches.

Cultivation Approaches/Photoperiods	Removal Efficiency (%)				Economic Efficiency (USD^−1^)			
	COD	TN	TP	CO_2_	COD	TN	TP	CO_2_
Culture 1								
12 h light:12 h dark	56.32 ^b^ ± 5.24	59.16 ^b^ ± 5.35	61.66 ^b^ ± 5.26	42.36 ^b^ ± 3.13	25.71 ^b^ ± 2.04	29.14 ^b^ ± 2.53	29.98 ^b^ ± 2.77	20.14 ^b^ ± 1.65
14 h light:10 h dark	63.43 ^a^ ± 5.76	68.22 ^a^ ± 5.26	70.06 ^a^ ± 5.24	48.87 ^a^ ± 3.96	30.05 ^a^ ± 2.28	32.25 ^a^ ± 3.02	33.63 ^a^ ± 2.61	24.38 ^a^ ± 2.19
16 h light:8 h dark	54.96 ^b^ ± 5.19	60.75 ^b^ ± 5.72	60.87 ^b^ ± 5.76	46.59 ^a^ ± 4.19	24.08 ^b^ ± 2.17	29.63 ^b^ ± 2.77	29.92 ^b^ ± 2.24	23.09 ^a^ ± 1.95
Culture 2								
12 h light:12 h dark	62.84 ^b^ ± 5.77	64.55 ^b^ ± 5.91	66.09 ^b^ ± 6.11	51.66 ^a^ ± 4.65	29.78 ^b^ ± 2.72	30.97 ^a^ ± 2.84	32.45 ^b^ ± 2.96	26.23 ^a^ ± 2.37
14 h light:10 h dark	70.24 ^a^ ± 6.86	72.99 ^a^ ± 6.12	73.83 ^a^ ± 5.81	52.26 ^a^ ± 5.37	34.13 ^a^ ± 3.01	36.42 ^a^ ± 3.25	37.16 ^a^ ± 3.07	27.37 ^a^ ± 2.71
16 h light:8 h dark	59.47 ^b^ ± 5.86	63.71 ^b^ ± 5.93	64.82 ^b^ ± 6.03	50.68 ^a^ ± 4.75	28.43 ^b^ ± 2.84	30.09 ^a^ ± 2.38	30.95 ^b^ ± 2.54	25.62 ^a^ ± 2.62
Culture 3								
12 h light:12 h dark	57.46 ^b^ ± 5.13	69.27 ^b^ ± 4.97	63.24 ^b^ ± 5.78	47.32 ^a^ ± 3.79	26.86 ^b^ ± 2.78	32.98 ^b^ ± 2.95	29.08 ^b^ ± 2.68	23.85 ^a^ ± 2.14
14 h light:10 h dark	68.54 ^a^ ± 5.96	74.25 ^a^ ± 5.95	71.98 ^a^ ± 6.04	49.94 ^a^ ± 4.92	32.63 ^a^ ± 2.35	37.83 ^a^ ± 3.16	35.68 ^a^ ± 2.64	24.59 ^a^ ± 2.77
16 h light:8 h dark	56.09 ^b^ ± 5.38	64.45 ^c^ ± 6.12	62.92 ^b^ ± 5.09	48.31 ^a^ ± 4.84	25.02 ^b^ ± 2.52	31.03 ^b^ ± 2.83	30.43 ^b^ ± 2.17	24.14 ^a^ ± 1.95

Note: Values with different superscript letters (e.g., ^a^,^b^,^c^) for the same cultivation approaches indicate a significant difference at *p* < 0.05 according to the Duncan’s multiple range tests.

**Table 5:** Mean values ± SD of the removal efficiency of biogas slurry nutrient and CO_2_ removal of light intensities for two cultivation approaches under the optimal photoperiods (14 h light:10 h dark).

Cultivation Approaches/Light Intensities	Mean Daily Productivity (g L^−1^ d^−1^)	COD Removal Efficiency (%)	TN Removal Efficiency (%)	TP Removal Efficiency (%)	CO_2_ Removal (%)
Culture 2					
250 μmol m^−2^ s^−1^	0.349 ± 0.021	79.12 ^b^ ± 4.38	80.13 ^a^ ± 6.25	82.06 ^a^ ± 6.35	62.83 ^a^ ± 4.11
450 μmol m^−2^ s^−1^	0.382 ± 0.024	83.43 ^a^ ± 5.74	81.27 ^a^ ± 6.77	83.52 ^a^ ± 6.61	59.37 ^b^ ± 5.07
650 μmol m^−2^ s^−1^	0.321 ± 0.018	72.86 ^c^ ± 5.87	76.48 ^b^ ± 5.91	81.57 ^a^ ± 5.97	57.49 ^b^ ± 5.79
Culture 3					
250 μmol m^−2^ s^−1^	0.336 ± 0.025	75.08 ^b^ ± 5.93	79.98 ^b^ ± 6.95	77.13 ^b^ ± 5.71	54.72 ^a^ ± 4.27
450 μmol m^−2^ s^−1^	0.368 ± 0.022	80.55 ^a^ ± 6.24	82.64 ^a^ ± 6.24	82.79 ^a^ ± 5.83	50.34 ^b^ ± 4.39
650 μmol m^−2^ s^−1^	0.314 ± 0.019	73.97 ^b^ ± 6.81	78.59 ^b^ ± 6.76	74.24 ^b^ ± 4.92	48.97 ^b^ ± 4.86

Note: Values with different superscript letters (e.g., ^a^,^b^,^c^) for the same cultivation approach indicate a significant difference at *p* < 0.05 according to the Duncan’s multiple range tests.
